# Substance Use Disorder as a Contributing Factor to Pregnancy-Associated Deaths

**DOI:** 10.1097/og9.0000000000000076

**Published:** 2025-04-03

**Authors:** Ryan Duggal, Amanda A. Allshouse, Maria Small, Marcela C. Smid

**Affiliations:** Department of Psychiatry and the Division of Maternal Fetal Medicine, Department of Obstetrics and Gynecology, University of Utah Health, Salt Lake City, Utah; the CDC Foundation, Atlanta, Georgia; and the Division of Maternal Fetal Medicine, Department of Obstetrics and Gynecology, Duke University Health System, Durham, North Carolina.

## Abstract

Substance use disorder (SUD) is increasingly linked with risk of accidental overdose and suicide among pregnant and postpartum people. However, less is understood about SUD as a contributing factor to natural manner of death among peripartum people. This study used National Vital Statistics System data to investigate differences in causes and timing of natural deaths between peripartum women and girls with and without SUD as a contributing factor to their deaths. Compared with deaths in individuals without SUD as a contributing factor, deaths in individuals with SUD as a contributing factor were less likely to occur postpartum and were more likely to be due to sepsis. These patterns have not been identified previously in the literature. Further investigation is needed to clarify the mechanisms behind SUD and causes of pregnancy-associated natural deaths.

## Abstract 2

Pregnancy-associated deaths in which substance use disorder was a contributing factor were significantly higher during pregnancy and disproportionately due to sepsis.

In the United States, substance use disorder (SUD) is an important contributing factor to pregnancy-associated deaths, most commonly postpartum accidental overdoses or suicides.^1,2^ However, SUD may also be an important contributing factor to severe maternal morbidity events and death.^3^ Our objective was to determine whether causes and timing of pregnancy-associated deaths from natural manner of death were different for individuals with compared with without SUD as a contributing factor.

## METHODS

We performed a population-based cohort study using data from the National Vital Statistics System (NVSS) (2015–2018). We included women and girls with *pregnancy-associated natural death*, defined as death occurring during pregnancy or within 1 year from the end of the pregnancy caused by disease or natural process. We excluded deaths with manners of death listed as accidental overdose, suicide, and homicide. Substance use disorder, specifically alcohol and illicit drug use during pregnancy and in the year postpartum, and cause of death were identified using International Classification of Diseases, Tenth Revision codes (Appendix 1, available online at http://links.lww.com/AOG/E64). In 2018, the NVSS changed the pregnancy coding system to capture maternal deaths, using a restricted age range of 10–44 years and an assigned maternal code to more accurately capture the underlying cause of death.^4^ We used this same approach to identify maternal deaths from 2015 to 2018. We compared age, race, and cause and timing of death (during pregnancy, 0–42 days postpartum, 43–365 days postpartum) between individuals with and without SUD as a contributing factor to their deaths using χ^2^ or Kruskal Wallis tests, as appropriate. Substance use disorder as a contributing factor was determined by the clinician completing the death certificate. For the two most common causes of death, we assessed the association between SUD as a contributing factor and timing of death in a multivariable logistic model with adjustment for age, race, and timing of death, with interaction testing between timing of death and SUD as a contributing factor to death. Statistical significance was defined as *P*<.05. All analyses were performed using R statistical software. Because this study was a retrospective chart review of de-identified data, the IRB of the University of Utah deemed it exempt.

## RESULTS

**Fig. 1.**
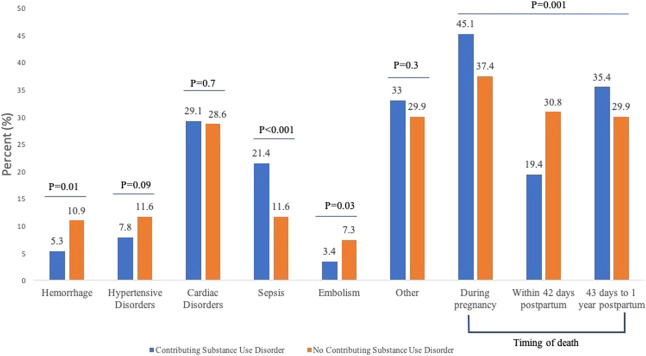
Causes and timing of pregnancy-associated death in individuals with contributing substance use disorder vs no contributing substance use disorder, National Vital Statistics Sample, 2015–2018.

Of 4,644 pregnancy-associated deaths, 4.4% (n=206) had SUD as a contributing factor. Groups with and without SUD differed by race (*P*<.001) but not age (Appendix 2, available online at http://links.lww.com/AOG/E64). Cause of death differed between individuals with and without SUD as a contributing factor (*P*=.01). Compared with individuals without SUD, those with SUD more frequently died of sepsis (21.4% vs 11.6%; *P*<.001) but less frequently died of hemorrhage (5.3% vs 10.9%; *P*=.01) or embolism (3.4% vs 7.3%; *P*=.03) (Fig. 1). There was no difference in causes of death from cardiac disorders (29.1% vs 28.6%; *P*=.90), hypertensive disorders (7.77% vs 11.6%; *P*=.09), or other or unknown causes of death (33.0% vs 30.0%; *P*=.3). Timing of death differed between those with and without SUD as a contributing factor: during pregnancy (45.1% vs 37.4%), within 42 days postpartum (19.4% vs 30.8%), and within 43 days to 1 year (35.4% vs 31.8%) (*P*=.002). There was no significant interaction between timing of death and SUD as a contributing factor in death caused by cardiac disorders (*P*=.44) or sepsis (*P*=.71). For the two most common causes of death, individuals who died of sepsis remained more likely to have a contributing factor of SUD (adjusted odds ratio 2.09, 95% CI, 1.46–2.93), whereas those who died of cardiac disease were no more likely to have SUD as a contributing factor (adjusted odds ratio 1.01, 95% CI, 0.91–1.39) (Table 1).

*Duggal. Substance Use Disorder and Pregnancy-Associated Deaths. O&G Open 2025*.

**Table 1.** Association Between Substance Use Disorder Status and Death Due to Cardiac Disease or Sepsis

Characteristic	Cause of Death			
	Sepsis		Cardiac Disease	
	Unadjusted OR (95% CI)	Adjusted OR (95% CI)*	Unadjusted OR (95% CI)	Adjusted OR (95% CI)*
SUD	2.13 (1.49–2.98)†	2.09 (1.46–2.93)†	1.12 (0.86–1.24)	1.01 (0.91–1.39)
Timing of death				
Pregnant at time of death	1	1	1	1
Death within 42 d postpartum	1.04 (0.84–1.29)	1.07 (0.86–1.32)	1.15 (0.99–1.32)	1.08 (0.93–1.26)
Death 43 d to 1 y postpartum	0.72 (0.57–0.92)†	0.71 (0.55–0.90)†	0.29 (0.24–0.35)†	0.27 (0.23–0.33)†
Age	0.99 (0.94–1.05)	0.98 (0.93–1.03)	1.76 (1.52–1.93)†	1.42 (1.31–1.53)†
Race				
American Indian/Alaska Native	0.67 (0.26–1.45)	0.58 (0.22–1.25)	1.37 (0.82–2.22)	1.30 (0.76–2.15)
Asian	0.79 (0.48–1.24)	0.82 (0.49–1.30)	1.12 (0.82–1.52)	1.13 (0.82–1.56)
Black	0.78 (0.63–0.96)†	0.79 (0.63–0.97)†	1.34 (1.17–1.54)	1.29 (1.11–1.48)
White	1	1	1	1

SUD, substance use disorder.

* Includes all characteristics presented in the table as covariates in the model.

† Indicates significance at *P*<.05.

## DISCUSSION

Among pregnancy-associated natural deaths, sepsis was disproportionately the cause of death and deaths occurred more frequently during pregnancy among individuals with SUD as a contributing factor. This pattern is distinctly different from accidental overdose and suicide, which disproportionately occur in the late postpartum period.^3,5^ The major strength of this study is our ability to distinguish SUD as a contributing factor to death from incidental presence of the diagnosis. A notable limitation in this study is the lack of the revised pregnancy checkbox for states before 2018, which may affect the accuracy of reporting maternal deaths. However, the restricted data curated by the NVSS for maternal deaths likely decreased ascertainment bias. This data set does not include information about comorbidities and other important confounders, limiting interpretation of results. The results of this study indicate that future efforts should focus on granular characterization of circumstances surrounding SUD-associated deaths, including natural deaths, overdoses, and suicides. These efforts should inform identification of potential interventions to prevent these deaths and stem the growing maternal mortality crisis in the United States.
